# Latent profile analysis of avoidant/restrictive food intake disorder in patients with inflammatory bowel disease in remission: A cross-sectional study

**DOI:** 10.1097/MD.0000000000049494

**Published:** 2026-06-26

**Authors:** Yuan Tian, Bo Gao, Jimin Qiao, Huanhuan Fei, Yin Wu, Mengya Xu

**Affiliations:** a Department of Gastroenterology, Nanjing First Hospital, Nanjing Medical University, Nanjing, China; b Department of Emergency, Nanjing First Hospital, Nanjing Medical University, Nanjing, China.

**Keywords:** avoidant/restrictive food intake disorder, fear-avoidance model, food-related quality of life, inflammatory bowel disease, latent profile analysis, visceral sensitivity index

## Abstract

Patients with inflammatory bowel disease (IBD) in remission experience a high incidence of avoidant/restrictive food intake disorder (ARFID) because of recurrent symptoms and long-term dietary management, which severely impair their quality of life. Varying degrees of ARFID exert differential effects on patients’ health status. However, the distribution characteristics of specific types of ARFID as well as their associated influencing factors among IBD patients in remission remain unclear and warrant further investigation. This study aims to explore the potential profiles of ARFID in patients with IBD in remission and to analyze the factors associated with these distinct potential profiles. A cohort of 239 participants was assembled for this cross-sectional investigation. These individuals, diagnosed with IBD and in clinical remission, were recruited from a specialized gastroenterology unit within a tertiary care facility located in Nanjing, China. The enrollment period spanned from June to September 2025. Utilizing convenience sampling, eligible patients were selected as research subjects following established methodological protocols. Binary logistic regression analysis revealed that patients who were employed full-time or reported a higher food-related quality of life were more likely to be classified into the High ARFID Symptom Risk Group than into the Low ARFID Symptom Risk Group, whereas patients with higher levels of gastrointestinal-specific anxiety were more likely to be classified into the Low ARFID Symptom Risk Group (*P* < .05). The results of the latent profile analysis revealed that the ARFID characteristics in IBD patients in remission could be categorized into 2 latent profiles: a High ARFID Symptom Risk Group (*n* = 139, 58.2%) and a Low ARFID Symptom Risk Group (*n* = 100, 41.8%). Health care professionals should prioritize identifying varying levels of ARFID among patients, with a particular focus on those who exhibit high-level ARFID (especially those who report feeling subjectively well but who demonstrate high-risk behaviors). It is essential to enhance the structured assessment of their dietary behaviors and implement targeted psychological and behavioral interventions to improve the capacity for identifying and addressing dietary-related risks, thereby alleviating these patients’ ARFID symptoms.

## 1. Introduction

Inflammatory bowel disease (IBD) is a group of chronic inflammatory intestinal disorders of unknown etiology, primarily comprising Crohn’s disease and ulcerative colitis. In recent years, the global incidence of IBD has continued to increase, and it is projected that the disease burden in China will further intensify by 2030.^[1]^ Dietary management constitutes a core component of self-care for patients with IBD in remission; appropriate dietary restrictions help control inflammation and reduce the risk of relapse.^[2]^ However, studies have shown that more than 90% of IBD patients restrict at least 1 food.^[3]^ Inappropriate or excessive restrictions may evolve into avoidant/restrictive food intake disorder (ARFID), a persistent eating disorder characterized primarily by an aversion to the sensory characteristics of food, a lack of interest in eating, or a fear of the consequences of eating, which can lead to nutritional imbalances, increased psychological burden, and a decline in quality of life.^[3,4]^

Currently, estimates of the prevalence of ARFID among patients with IBD vary widely (11%–53%), as many studies have failed to strictly distinguish between periods of active disease and remission.^[4]^ According to the Diagnostic and Statistical Manual of Mental Disorders, Fifth Edition (DSM-5) criteria, dietary avoidance that is directly attributable to active IBD should be considered an adaptive response rather than ARFID.^[5,6]^ Research confirms that gastrointestinal-specific anxiety is an associated factor of ARFID in patients with IBD in remission.^[7]^ Current domestic research largely focuses on patients in the active phase of the disease, paying insufficient attention to those in remission. Although some studies have reported that food literacy serves as an associated factor of ARFID in patients with active IBD,^[8]^ dietary avoidance behaviors in patients in remission tend to be more subtle. Moreover, they may engage in complex interactions with factors such as gastrointestinal-specific anxiety and quality of life. Furthermore, the clinical manifestations of ARFID frequently overlap with IBD symptoms, thereby increasing the difficulty of both diagnosis and management. Dietary avoidance in active IBD is predominantly an adaptive disease-related behavior, whereas persistent avoidance during remission is far more likely to progress to pathological ARFID. This population has an extremely low clinical identification rate and represents a critical gap in long-term IBD management.

The fear-avoidance model (FAM) offers a crucial theoretical perspective for understanding the persistence of ARFID. According to this model, individuals engage in avoidance behaviors driven by a fear of symptom recurrence; while this provides short-term anxiety relief, in the long-term, through the mechanism of negative reinforcement, it entrenches avoidance behaviors into a self-sustaining pathological pattern.^[9]^ In patients with IBD, symptoms such as abdominal pain and diarrhea can trigger gastrointestinal-specific anxiety, prompting patients to avoid suspected foods; subsequently, owing to a lack of corrective safety experiences, this avoidance generalizes to a broader range of items, ultimately leading to the development of ARFID.^[10]^

Drawing upon the FAM framework, ARFID presents with heterogeneous categorical expressions in individuals diagnosed with IBD. The application of Latent profile analysis, a method oriented towards individual-based typology, facilitates the delineation of covert ARFID subgroups. This differentiation establishes a critical premise for formulating tailored therapeutic protocols. Accordingly, the present investigation seeks to delineate the typological characteristics of ARFID among remitted IBD patients through latent profile analysis, concurrently exploring pertinent correlative determinants. These findings are intended to furnish an empirical basis for enhancing clinical screening of elevated-risk cohorts and refining subsequent psychosocial and behavioral management approaches.

## 2. Methods

### 2.1. Research design and participant selection

A power analysis was conducted a priori to determine the requisite sample size. Employing the single-proportion formula applicable to cross-sectional designs: *n* = Z^2^P(1 − P)/d^2^_,_^[11]^ the calculation incorporated a previously reported ARFID prevalence estimate of 15.4% within the IBD population.^[12]^ With a two-sided significance level (α) of 0.05 (*Z* = 1.96) and a permissible margin of error (d) set at 0.05, a minimum of 201 participants was deemed necessary. Anticipating a potential 10% to 15% rate of nonresponse or incomplete data, the target recruitment range was established at 224 to 237 cases. The final analytical sample comprised 239 valid responses. These individuals, diagnosed with IBD and in clinical remission, were recruited from a specialized gastroenterology unit within a tertiary care facility located in Nanjing, China. The enrollment period spanned from June to September 2025. Utilizing convenience sampling, eligible patients were selected as research subjects following established methodological protocols. Eligibility was contingent upon several criteria: a definitive IBD diagnosis confirmed through clinical, radiographic, and endoscopic evaluation^[13]^; attainment of clinical remission, operationally defined as a partial Mayo score ≤2 accompanied by a rectal bleeding subscore of 0 for ulcerative colitis, or a Harvey–Bradshaw index <5 combined with biochemical markers (C-reactive protein <5 mg/L and fecal calprotectin <150 μg/g) for Crohn’s disease^[14]^; a documented disease history exceeding 6 months; and age of 18 years or older, possessing adequate literacy for questionnaire comprehension. Exclusionary conditions encompassed: concurrent medical or psychiatric diagnoses exerting significant influence on dietary patterns (e.g., irritable bowel syndrome, poorly managed diabetes, severe psychological disorders); recent utilization of enteral or parenteral nutritional support; adherence to restrictive long-term dietary regimens (such as ketogenic or strict vegan diets); and current pregnancy or lactation status. All enrolled individuals provided digital informed consent and completed the assessment battery anonymously via a QR code-linked platform (Wenjuanxing). The investigation received formal ethical endorsement from the institutional review board (Approval No.: KY20250624-KS-01).

### 2.2. Instrumentation and data acquisition

#### 2.2.1. Sociodemographic and clinical characteristics survey

An instrument was devised to capture key participant attributes. Its development was informed by extant literature^[12]^ and finalized through research team consensus. This tool gathered information across 2 domains: Sociodemographic variables, including gender, age, educational attainment, marital and employment status, residential location, health insurance type, average monthly household income per capita, religious affiliation, body mass index, and domestic living situation. Clinical variables pertinent to IBD, encompassing specific diagnosis (ulcerative colitis vs Crohn’s disease), illness duration, disease activity status, occurrence of complications, and pharmacological treatments employed.

#### 2.2.2. Assessment of ARFID phenotypes

Participants’ symptom profiles pertaining to ARFID were quantified employing a sinicized iteration^[15]^of the Nine-Item ARFID Screen (NIAS).^[16]^ This assessment tool encompasses 9 discrete items, systematically allocated to 3 distinct phenotypic dimensions: pickiness in food selection (items 1–3), absence of sustained appetite (items 4–6), and distress associated with ingestion (items 7–9). Each statement is evaluated utilizing a Likert-type response framework spanning 6 gradations (0 denoting “complete disagreement” to 5 representing “total agreement”), thereby producing cumulative scores ranging from 0 to 45 points. Higher total values on this metric correlate with an increased propensity for ARFID manifestation. Scholarly precedent has established a threshold value of 28 points or above as suggestive of elevated clinical risk for this condition.^[7]^ Within the current sample, the scale exhibited outstanding internal consistency, with a Cronbach’s alpha coefficient reaching 0.860. The NIAS is a validated screening tool for ARFID, with a score ≥28 indicating high clinical risk of ARFID. This study did not perform-aligned structured clinical interviews to confirm a formal ARFID diagnosis; all findings are specific to ARFID symptom risk, not confirmed disease.

#### 2.2.3. Assessment of psychological distress indices

The psychological constructs of anxiety and depression were quantified utilizing the Patient Health Questionnaire-4 (PHQ-4)^[17]^ originally designed this tool, whose reliability was later corroborated by Adzrago et al.^[18]^ This brief measure integrates 4 queries divided into distinct dimensions: the initial 2 address anxiety, while the subsequent 2 pertain to depression. Respondents indicate the prevalence of symptoms experienced over the past 2 weeks by selecting options on a 4-point Likert scale, ranging from 0 (denoting no symptoms) to 3 (indicating near-daily occurrence). The composite scoring range extends from 0 to 12, wherein ascending totals directly correlate with an intensification of distress severity. Applying established clinical benchmarks, a separate score of ≥3 on either subscale signals a probable diagnosis for the corresponding disorder. The instrument demonstrated exemplary internal reliability in the current analysis, yielding a Cronbach’s alpha coefficient of 0.904.

#### 2.2.4. Assessment of gastrointestinal-specific anxiety

The measurement of anxiety specific to the gastrointestinal domain employed the Visceral Sensitivity Index (VSI). This psychometric tool, whose development is credited to Labus et al^[19]^ and whose psychometric properties were subsequently substantiated by Rizzo et al,^[20]^ comprises 15 items that collectively evaluate 5 principal dimensions: worry, fear, vigilance, sensitivity, and avoidance. Participant responses are recorded along a 6-point Likert scale, anchored by the poles “strongly disagree” (0) and “strongly agree” (5), inclusive of multiple inversely-scored items. The aggregate score, potentially spanning from 0 to 75, demonstrates a positive association with the severity of gastrointestinal-centric anxiety. Within the current sample, the instrument exhibited excellent internal consistency, evidenced by a Cronbach’s α value of 0.931.

#### 2.2.5. Evaluation of food-related quality of life

The Food-Related Quality of Life Scale (FRQoL-25)^[21]^was employed within a Chinese cohort to evaluate the psychosocial sequelae associated with dietary patterns. Originally designed by Hughes and colleagues,^[22]^ this instrument comprises 25 unidimensional items that assess multiple dimensions including the affective experience of mealtimes and the dynamics of commensality. Participant responses are captured utilizing a 5-point Likert scale anchored at “strongly agree” and “strongly disagree.” Subsequent to the inversion of designated item scores, cumulative results span from 25 to 125, with elevated aggregate values signifying a more positive experiential state concerning food. For the present investigation, the scale exhibited exceptional reliability in its internal consistency (Cronbach’s α = 0.979).

#### 2.2.6. Assessment of food literacy competency

The competency in food literacy was evaluated through the implementation of a questionnaire originally developed in the Chinese language by Qian and colleaguesl.^[23]^ This measurement tool, consisting of fifteen items, delineates 3 distinct constituent domains: planning and management related to food (seven items), selection of food items (three items), and preparatory attitudes concerning food (five items). Utilizing a 4-point Likert response framework where 1 denotes “never” and 4 indicates “always,” the instrument generates an aggregate score spanning 15 to 60 points. Higher composite scores are directly indicative of superior competency levels in food literacy. For the present study, the internal consistency of this scale demonstrated excellent reliability, evidenced by a Cronbach’s alpha coefficient of 0.942.

### 2.3. Analytical methodology

Statistical processing utilized SPSS 26.0 alongside Mplus 8.3. Categorical measures were denoted as frequencies and percentages; comparative analyses across groups relied on chi-square or Fisher’s exact testing, contingent upon appropriateness. For continuous variables adhering to normality, results were delineated as mean ± standard deviation; cross-group assessments employed either independent-samples *t* tests or ANOVA. Non-parametric continuous measures were characterized by their median and interquartile range, with inter-group distinctions examined through the Mann–Whitney *U* test.

Latent Profile Analysis was executed within Mplus 8.3, where the 9 NIAS item scores functioned as manifest indicators for model estimation spanning 1 to 5 latent classes. The relative adequacy of contending models was ascertained through a multi‑metric assessment framework, incorporating the Akaike Information Criterion (AIC), Bayesian Information Criterion (BIC), its adjusted counterpart (adjusted BIC), entropy, as well as the Lo‑Mendell‑Rubin adjusted likelihood ratio test and the bootstrap likelihood ratio test. Model selection was guided by the following pre‑specified criteria: statistical significance of the LMRT (*P* < .05) and bootstrap likelihood ratio test (*P* < .05) to justify adding a class; higher entropy (closer to 1) indicating clearer class separation; lower BIC values suggesting better parsimony; and average posterior probabilities (AvePP) of at least 0.90 for each class, denoting high classification accuracy. In addition, class solutions containing a subgroup comprising <5% of the total sample were considered statistically unstable and were rejected. The definitive profile structure was determined by a synthesized appraisal of these diagnostic indices, with a strong emphasis on selecting a solution that offered both statistical robustness and clear clinical interpretability (i.e., distinct, meaningful profiles relevant to patient management).

Subsequent to profile derivation, binomial logistic regression analyses were conducted to ascertain factors associated with membership allocation among the resultant latent classes.The setting of the reference group is shown in Table 1. For the binary logistic regression analysis, the forced entry (Enter) method was used. All variables listed in Table 2 including the 3 primary predictors (employment status, FRQoL‑25, VSI), the 6 pre‑specified core confounders (IBD subtype, disease duration, height and weight, history of intestinal surgery, presence of disease complications, number of concurrent medications), and all other sociodemographic, clinical, and psychological covariates were simultaneously entered into the model, irrespective of their statistical significance in univariate analyses. A two-tailed alpha threshold of 0.05 was established for all inferential procedures. All survey‑derived data integrated within these analytical stages were fully complete, exhibiting no instances of missingness.

**Table 1:** Variable assignment.

Variable	Variable assignment
Gender	0 = Male, 1 = Female
Education level	0 = Primary school or below, 1 = Junior high school, 2 = Senior high school or technical secondary school, 3 = Associate degree or bachelor’s degree, 4 = Graduate degree or above
Marital status	0 = Unmarried, 1 = Married/cohabiting, 2 = Divorced, 3 = Widowed
Employment status	0 = Full-time employee, 1 = Non-full-time
Domicile	0 = City, 1 = County/town, 2 = Rural area
Medical payment method	0 = Medical insurance or public funding, 1 = Self-paid
Per capita monthly household income	0 = Below 3000 RMB/mo, 1 = 3000–5000 RMB/mo, 2 = 5001–8000 RMB/mo, 3 = 8001–10,000 RMB/mo, 4 = Above 15,000 RMB/mo
Religion	0 = No religious belief, 1 = Buddhism, 2 = Taoism, 3 = Christianity, 4 = Other
Diagnosis	0 = Ulcerative colitis, 1 = Crohn’s disease
Course	0=≤1 yr, 1 = 1–5 yrs, 2 = 5–10 yrs, 3=≥10 yrs
Complications	Complications
Number of medication types	0 = 1 type, 1 = 2–3 types, 2 = More than 3 types

Variables coded as 0 serve as the reference category.

**Table 2:** Binary logistic regression analysis of potential ARFID group types in patients with remitted IBD (*n* = 239).

Unadjusted							
Variable	*ß*	S.E.	*z*	Wald χ2	*P*	OR	95% CI for OR
Constant	0.155	1.038	0.150	0.022	0.881	1.168	0.153 ~ 8.932
Sex	0.654	0.499	1.309	1.713	0.191	1.922	0.722 ~ 5.116
Age	−0.013	0.018	−0.754	0.569	0.451	0.987	0.953 ~ 1.022
Degree of education	−0.114	0.196	−0.581	0.338	0.561	0.893	0.608 ~ 1.310
Marital status	−0.176	0.353	−0.500	0.250	0.617	0.838	0.420 ~ 1.675
Not in full-time employment	−0.185	0.089	−2.088	4.360	0.037	0.831	0.698 ~ 0.989
Domicile	−0.324	0.245	−1.323	1.750	0.186	0.724	0.448 ~ 1.169
Medical payment methods	0.616	0.777	0.793	0.628	0.428	1.851	0.404 ~ 8.480
Per capita monthly household income	−0.097	0.151	−0.645	0.416	0.519	0.908	0.676 ~ 1.219
Religion	−0.131	0.176	−0.747	0.558	0.455	0.877	0.621 ~ 1.238
Height	0.036	0.032	1.138	1.294	0.255	1.037	0.974 ~ 1.103
Weight	0.016	0.015	1.030	1.061	0.303	1.016	0.986 ~ 1.046
Living status	0.305	0.514	0.594	0.353	0.553	1.357	0.495 ~ 3.720
Diagnosis	0.024	0.412	0.058	0.003	0.954	1.024	0.457 ~ 2.297
Course	0.272	0.200	1.362	1.854	0.173	1.313	0.887 ~ 1.942
Complications	0.383	0.354	1.083	1.172	0.279	1.467	0.733 ~ 2.937
Number of medication types	−0.345	0.238	−1.449	2.098	0.147	0.708	0.444 ~ 1.129
Food literacy	−0.017	0.023	−0.737	0.543	0.461	0.983	0.939 ~ 1.029
FRQoL	0.040	0.014	2.861	8.186	0.004	1.041	1.013 ~ 1.070
Self-efficacy	−0.006	0.010	−0.624	0.390	0.532	0.994	0.974 ~ 1.014
PHQ-4	−0.043	0.071	−0.596	0.356	0.551	0.958	0.833 ~ 1.102
VSI	−0.032	0.013	−2.394	5.732	0.017	0.968	0.943 ~ 0.994
**Adjusted**							
**Variable**	**ß**	**S.E.**	**z**	**Waldχ2**	**p**	**OR**	**95% CI for OR**
Constant	0.155	1.038	0.150	0.022	0.881	1.168	0.153 ~ 8.932
Not in full-time employment	−0.142	0.075	−1.887	3.562	0.059	0.868	0.749 ~ 1.005
FRQoL	0.040	0.013	3.111	9.676	0.002	1.041	1.015 ~ 1.067
VSI	−0.038	0.011	−3.604	12.987	0.000	0.963	0.943 ~ 0.983

The Low ARFID Symptom Risk Group served as the reference category for the dependent variable. All variables were entered using the forced entry (Enter) method; inclusion did not depend on univariate significance. Hosmer-Lemeshow goodness-of-fit test: χ^2^ = 3.544, df = 8, *P* = .896. Reference groups for categorical variables are indicated in parentheses.

The setting of the reference group is shown in Table 1.

Unadjusted: All variables were simultaneously entered into the regression model.

Adjusted: Variables with *P* < .05 in one-way ANOVA were included.

FRQoL = food-related quality of life, PHQ-4 = patient health questionnaire-4, VSI = visceral sensitivity index.

## 3. Results

### 3.1. General information on the survey subjects

A total of 239 patients were included in the study. As shown in Table 3, there were no significant differences between the 2 groups in terms of sex (male) (61.9% vs 61.0%, *P* = .891), age (38 [30, 47] vs 40 [32, 49], *P* = .258), and food literacy (34.57 ± 8.00 vs 36.32 ± 7.92, *P* = .095). However, significant differences were observed in full-time employment (66.9% vs 51.0%, *P* = .013), weight (61 [54, 71] vs 57 [51, 65], *P* = .021), complications (28.1% vs 41.0%, *P* = .036), number of medications (≥3) (9.4% vs 18.0%, *P* = .049), food-related quality of life (62.27 ± 14.21 vs 52.73 ± 11.20, *P* < .001), PHQ-4 (7 [5, 8] vs 8 [7, 10], *P* < .001), and VSI (41.17 ± 14.78 vs 52.72 ± 15.24, *P* < .001).

**Table 3:** Univariate analysis of the latent profiles of ARFID in patients with remitted IBD (*n* = 239)

Variable	High ARFID symptom risk group (*n* = 139)	Low ARFID symptom risk group (*n* = 100)	Statistic	*P*
Demographics				
Sex (Male), *n* (%)	86 (61.9)	61 (61.0)	χ^2^ = 0.019	0.891
Age (yr), Mdn (IQR)	38 (30, 47)	40 (32, 49)	Z = −1.131	0.258
Full-time employment, *n* (%)	93 (66.9)	51 (51.0)	χ^2^ = 6.144	0.013
Clinical characteristics				
Weight (kg), Mdn (IQR)	61 (54, 71)	57 (51, 65)	Z = −2.309	0.021
Complications (Yes), *n* (%)	39 (28.1)	41 (41.0)	χ^2^ = 4.375	0.036
Number of medications (≥3), *n* (%)	13 (9.4)	18 (18.0)	χ^2^ = 3.853	0.049
Psychosocial variables				
Food literacy, M (SD)	34.57 ± 8.00	36.32 ± 7.92	*t* = −1.678	0.095
FRQoL, M (SD)	62.27 ± 14.21	52.73 ± 11.20	*t* = 5.578	<0.001
PHQ-4, Mdn (IQR)	7 (5, 8)	8 (7, 10)	Z = −3.797	<0.001
VSI, M (SD)	41.17 ± 14.78	52.72 ± 15.24	*t *= -5.883	<0.001

*χ*^2^: Chi-square test; Z: Mann–Whitney test; *t*: *t*-test.

M = Mean, SD = Standard deviation; Mdn = Median, IQR = interquartile range; FRQoL = food-related quality of life, PHQ-4 = patient health questionnaire-4, VSI = visceral sensitivity index.

Continuous variables are presented as M (SD) or Mdn (IQR) based on their distribution.

### 3.2. Results of latent profile analysis of ARFID in patients with IBD in remission

**Figure 1.**
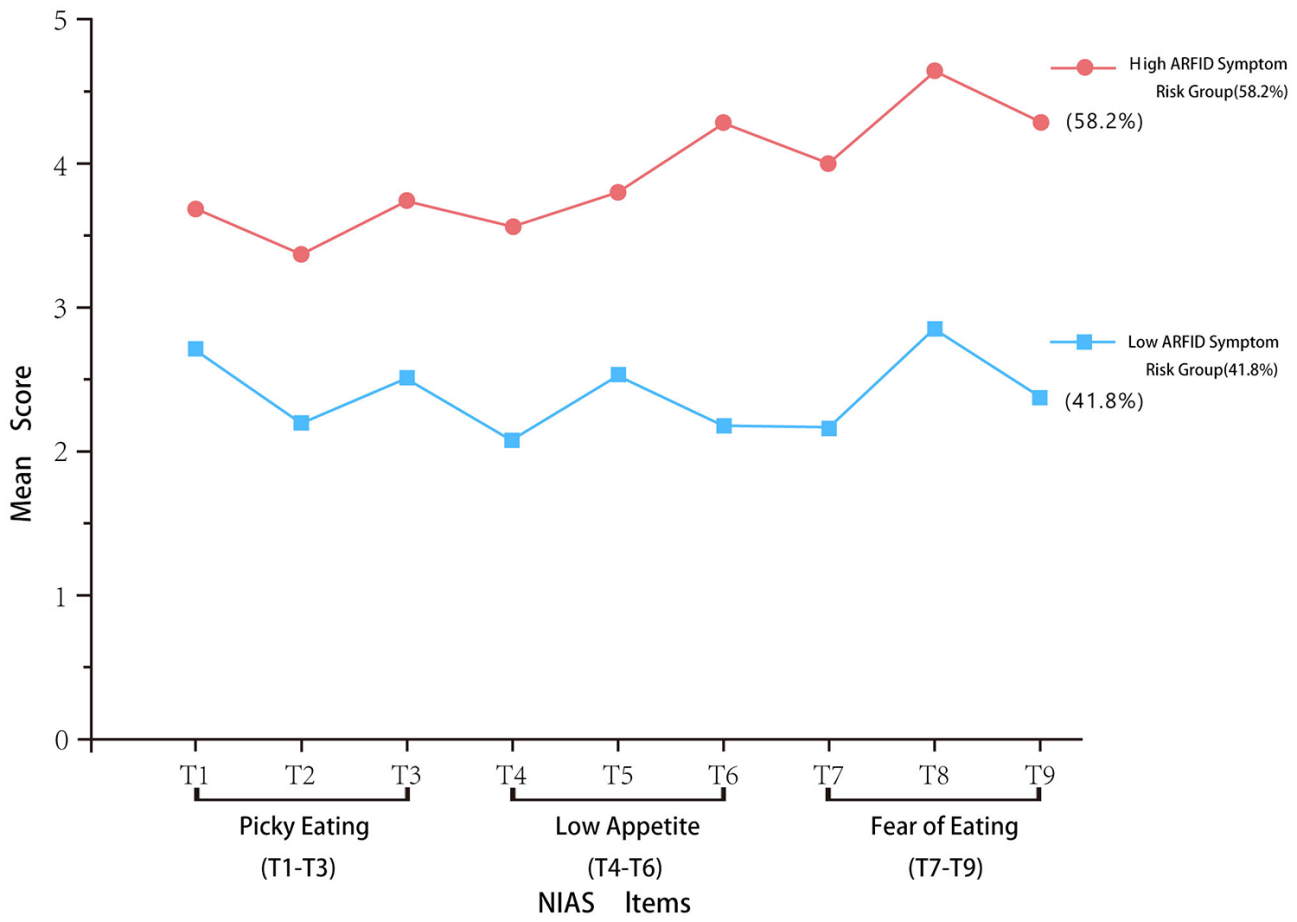
Distribution of 2 latent profiles of ARFID in patients with remitted IBD. Line graph showing mean scores for the low ARFID symptom risk group (41.8% of patients) and high ARFID symptom risk group (58.2% of patients) across all 9 items of the NIAS. The high ARFID symptom risk group shows elevated scores across all 3 domains: picky eating (items 1–3), poor appetite (items 4–6), and fear of eating (items 7–9). ARFID = avoidant/restrictive food intake disorder, IBD = inflammatory bowel disease, NIAS = nine-item ARFID screen.

Utilizing the 9 items of the NIAS as indicators, latent profile models encompassing 1 to 5 classes were evaluated. Model fit indices AIC, BIC, and adjusted BIC demonstrated a consistent decline with the incremental addition of classes. Model selection was based on LMRT significance (*P* < .05), AvePP (≥0.90), entropy, and BIC, while also considering class size (≥5% of the sample) and clinical distinctiveness. The 2‑class solution was retained because it was the most parsimonious model with a significant LMRT (*P* < .001), all classes meeting the AvePP ≥ 0.90 criterion, and clear clinical interpretability. In contrast, the 3‑class solution had a non‑significant LMRT (*P* = .368); the 4‑class solution contained a class with AvePP = 0.892 (<0.90) and lower entropy; and the 5‑class solution had a non‑significant LMRT (*P* = .194) plus 2 classes with proportions <5%. Therefore, the 2‑class solution was chosen as the optimal balance of statistical fit and clinical utility. This analysis delineated 2 distinct subgroups: a High ARFID Symptom Risk Group (58.2% of the sample) and a Low ARFID Symptom Risk Group (41.8%). The specific fit indices for each model are detailed in Table 4. See Figure 1. To validate the stability of the 2‑class solution, a sensitivity analysis was performed by increasing the number of random starts in Mplus 8.3 from STARTS 500 100 to STARTS 2000 500. All model fit indices (loglikelihood, AIC, BIC, entropy, class proportions, AvePP, and item means) remained identical to the original model. Moreover, all 500 final optimizations converged to the same global log‑likelihood solution, confirming excellent stability and robustness of the 2‑class structure.

**Table 4:** Fit index of the latent profile model of ARFID in patients with remitted IBD (*n* = 239).

MODEL	AIC	BIC	aBIC	Entropy	LMRT P	BLRT P	Class proportions	AvePP
1	7534.411	7596.987	7539.932	–	–	–	1	
2	7027.922	7125.263	7036.511	0.862	<0.001	<0.001	0.58159, 0.41841	0.955, 0.965
3	6947.830	7079.935	6959.486	0.838	0.368	<0.001	0.46025, 0.14226, 0.39749	0.950, 0.914, 0.908
4	6865.350	7032.221	6880.074	0.828	0.041	<0.001	0.43515, 0.22176, 0.21339, 0.12971	0.966, 0.934, 0.912, 0.892
5	6828.540	7030.175	6846.331	0.839	0.194	<0.001	0.30097, 0.15696, 0.15074, 0.24366, 0.14766	0.958, 0.911, 0.913, 0.911, 0.940

The 2-class model was selected as the final model based on the overall assessment of fit, interpretability, class size, and solution stability. Likelihood ratio test results are reported for completeness but were not used as sole criteria for class enumeration. AvePP = average posterior probability; values ≥0.90 indicate high classification accuracy.

AIC = Akaike information criterion, BIC = Bayesian information criterion, aBIC = adjusted Bayesian information criterion, LMRT = Lo-Mendell-Rubin adjusted LRT test, BLRT = a bootstrap-based likelihood ratio test.

### 3.3. Univariate analysis of potential ARFID profiles in patients with IBD in remission

Comparative analysis between the 2 identified ARFID latent profiles revealed statistically significant disparities (*P* < .05) across multiple domains. Specifically, significant differences were observed in body weight metrics, the prevalence of complications, polypharmacy (use of 3 or more medications), food-related quality of life (FRQoL-25), psychological distress (PHQ-4), and gastrointestinal-specific anxiety (VSI). A comprehensive summary of these comparisons is presented in Table 3.

### 3.4. Binary logistic regression analysis of potential ARFID profiles in patients with IBD in remission

A binary logistic regression analysis was conducted to identify factors associated with ARFID latent profile membership, with the Low ARFID Symptom Risk Group serving as the reference category. All variables presented in Table 2 were simultaneously entered using the forced entry (Enter) method, regardless of their univariate significance. These included: the 3 primary independent variables of interest (employment status, FRQoL‑25, VSI); 6 core confounding variables (IBD subtype, disease duration, height and weight history of intestinal surgery, presence of disease complications, number of concurrent medications); and all other sociodemographic and clinical covariates (e.g., age, sex, education, marital status, residence, income, religion, living status, food literacy, self‑efficacy, PHQ‑4, etc).

Table 2 presents the multivariable adjusted odds ratios (ORs) with 95% confidence intervals from the final forced‑entry model. After mandatory adjustment for the 6 core confounders, the results were as follows: Full‑time employment was significantly associated with a higher likelihood of being in the High ARFID Symptom Risk Group (adjusted OR = 0.831, 95% CI: 0.698–0.989, *P* = .037). Note: The OR <1 reflects coding where full‑time = 0, non‑full‑time = 1; thus, full‑time employment increases the risk of high ARFID classification. Higher food‑related quality of life (FRQoL‑25) remained significantly associated with the High ARFID Symptom Risk Group (adjusted OR = 1.041, 95% CI: 1.013–1.070, *P* = .004). Higher gastrointestinal‑specific anxiety (VSI) remained significantly associated with the Low ARFID Symptom Risk Group (adjusted OR = 0.968, 95% CI: 0.943–0.994, *P* = .017); for example, lower VSI was linked to the high‑risk group. None of the 6 mandatory confounding variables reached statistical significance in the fully adjusted model. The model’s calibration was assessed using the Hosmer‑Lemeshow goodness‑of‑fit test, which yielded χ^2^ = 3.544 (df = 8, *P* = .896), indicating no significant difference between predicted and observed probabilities and confirming good model fit. Detailed regression coefficients for all variables are provided in Table 2.

## 4. Discussion

### 4.1. Two potential profiles of ARFID in IBD patients in remission

Through latent profile analysis, this study identified 2 categories of ARFID among IBD patients in remission, a High ARFID Symptom Risk Group (58.2%) and a Low ARFID Symptom Risk Group (41.8%), indicating that significant heterogeneity in dietary avoidance behaviors persists among patients in remission.

The High ARFID Symptom Risk Group scored higher across all NIAS dimensions but paradoxically exhibited lower gastrointestinal-specific anxiety (VSI) and higher food-related quality of life (FRQoL-25). Analyzed through the lens of the FAM, these findings reveal the negative reinforcement mechanism of avoidance behavior: patients avoid food because of a fear of symptoms, and successful avoidance alleviates anxiety, thereby reinforcing the cognition that only strict dietary control can prevent symptoms.^[24,25]^ Over time, fear generalizes, and dietary rules become rigid. However, owing to the sense of control gained from short-term “safe experiences, anxiety decreases, and the behavior transitions from being “fear driven to “habitual avoidance.”^[26]^ Full-time work reinforces this pattern professional pressures amplify the short-term benefits of avoidance (keeping the job), leading to its entrenchment.^[27]^ Clinicians should be vigilant regarding working patients who report feeling well (low anxiety, high satisfaction with their diet) and should look beyond self-reports to conduct structured behavioral assessments.

The Low ARFID Symptom Risk Group had lower NIAS scores but higher VSI scores and lower FRQoL-25 scores. According to the FAM, these patients are in the early stage of fear-avoidance: they are highly vigilant about the consequences of eating, and anxiety directly affects their eating experience; however, avoidance has not yet become entrenched and remains linked to specific symptoms.^[28]^ Health care providers should encourage patients to express their concerns about eating and help them develop a flexible understanding of the relationship between their symptoms and eating habits, thereby preventing the development of rigid thinking.

In summary, this study identifies a subgroup of IBD patients in remission characterized by “subjective well-being but high-risk behavior,” which represents a classic manifestation of the FAM negative reinforcement mechanism. Relying solely on patient reports may lead to the misdiagnosis of high-risk individuals; therefore, behavioral screening and psychosocial factors must be integrated into the assessment.

### 4.2. Factors influencing potential ARFID profiles in IBD patients in remission

This study further identified correlated factors for different ARFID groups. The analysis revealed that full-time employment and higher food-related quality of life (FRQoL-25) were independently associated factors of assignment to the High ARFID Symptom Risk Group, whereas higher gastrointestinal-specific anxiety (VSI) was independently associated with assignment to the Low ARFID Symptom Risk Group. Although these results may seem contradictory, they reveal a specific phenomenon of “high-functioning avoidance” among IBD patients, which is consistent with the findings of Grossberg et al^[7]^ and Tu et al^[12]^ Although these patients meet the screening criteria for ARFID, they do not exhibit typical functional impairments (such as social avoidance or reduced quality of life); instead, they appear to be “superficially well adjusted” in terms of occupational and diet-related life satisfaction. The underlying mechanisms can be summarized as follows:

First, from a behavioral perspective, the mechanism of negative reinforcement reinforces avoidance behavior. To maintain the normal rhythm of their full-time job, patients use strict dietary control to avoid unpredictable gastrointestinal symptoms. This strategy is “effective” in the short-term, allowing them to avoid postprandial discomfort (negative reinforcement), thereby forming “habitual avoidance.” At this point, these patients’ low VSI and high FRQoL-25 score stem not from the elimination of fear but from the success of avoidance behavior that is, achieving temporary peace by completely distancing themselves from the “threat.”^[24]^

Second, from a neurobiological perspective, visceral hypersensitivity amplifies the perception of intestinal signals as threatening. Patients with IBD in remission may still exhibit low-grade inflammation or visceral hypersensitivity. Even when objective inflammatory markers are normal, patients have a lower threshold for perceiving normal physiological signals, making them prone to misinterpreting ordinary intestinal motility as a sign of disease recurrence. This “hypervigilance–misinterpretation–avoidance” cycle causes avoidance behaviors to become detached from the objective clinical condition, leading to rigidity and persistence.^[29]^

Furthermore, the gut microbiota–gut–brain axis may play a role in maintaining this vicious cycle. The diversity of the gut microbiota in IBD patients in remission typically remains low. Long-term food avoidance leads to reduced dietary fiber intake, which further exacerbates microbial dysbiosis. The resulting metabolites influence central nervous system function via the vagus nerve and immune pathways, which in turn exacerbate visceral hypersensitivity and the tendency to interpret gut signals as threatening. This creates a “food avoidance microbiota dysbiosis visceral hypersensitivity behavioral entrenchment” closed loop, providing a biological basis for understanding the “behavior–perception dissociation” observed in the High ARFID Symptom Risk Group.^[30]^

In summary, the “low anxiety + high quality of life” observed in the High ARFID Symptom Risk Group represents a fragile adaptive phenomenon. This quality of life comes at the cost of reduced dietary diversity, increased cognitive load, and potential nutritional risks.^[31]^ The fact that a patient feels well does not necessarily mean that he or she is behaving safely. Therefore, health care providers should be vigilant with respect to “high-functioning avoidance” patients and use structured interviews and behavioral screening tools (such as the NIAS) for early identification. In terms of intervention, the cognitive-behavioral therapy for ARFID framework can be utilized.^[32]^ Through psychological education, we can help patients understand that “feeling good does not necessarily mean behaving safely.” By utilizing graded exposure and gradual food reintroduction, we can help them break the fear-avoidance cycle and rebuild a flexible relationship with food.^[33]^

### 4.3. The bidirectional role of gastrointestinal-specific anxiety in ARFID among IBD patients in remission

This study revealed that patients with high VSI scores were more likely to be classified into the Low ARFID Symptom Risk Group, which is consistent with the findings of previous research.^[7,34]^ According to the results of the FAM analysis, moderate gastrointestinal-specific anxiety may serve as an “adaptive warning signal”: it helps patients remain aware of the “food–symptom” association and distinguish between foods that they cannot tolerate and safe foods rather than avoiding all foods.^[35,36]^ Although patients in the Low ARFID Symptom Risk Group experience higher levels of anxiety, their avoidance behaviors are not rigid, and their eating behaviors retain flexibility, leaving room for intervention.

Conversely, a low VSI associated with the High ARFID Symptom Risk Group warrants vigilance against “disconnected warning signals.” This represents not a healthy gut–brain interaction but a long-term avoidance that disrupts feedback loops: by completely eliminating trigger situations, patients experience reduced subjective anxiety but lose the ability to discern “which foods are truly harmful,” leading to rigid, generalized restriction patterns.^[37,38]^ Restrictive eating behaviors are reinforced by the absence of internal distress signals, thereby increasing hidden risks. In other words, ARFID patients with low VSI scores are not “free from anxiety”; rather, they “freeze” their anxiety at the behavioral level through complete avoidance.^[39]^

In clinical practice, it is important to recognize the value of a bidirectional assessment of the VSI. For individuals with a high VSI score (the “fear-active” stage of the FAM), structured food journaling and personalized food exploration should be guided with empathy to prevent the generalization of anxiety.^[40]^ For working patients who report positive subjective experiences (low VSI and high FRQoL-25), health care providers should be vigilant about the possibility that these patients may enter an “avoidance-fixation” phase, and they should proactively use behavioral screening tools to identify behavioral–perceptual dissociation.^[41]^ In nursing education, nurses should explain to patients the warning significance of moderate anxiety, help them understand the long-term costs of avoidance behaviors, and, with professional support, implement a gradual food reintroduction process to restore dietary diversity and a healthy perception–behavior feedback loop.^[42,43]^ At the same time, nurses can guide patients in keeping a food-symptom diary to rebuild a realistic perception of the causal relationship between food and symptoms, thereby breaking the vicious cycle of fear and avoidance.

This is a cross-sectional study, which cannot establish the temporal sequence or causal direction between low gastrointestinal-specific anxiety and high ARFID behaviors. Prospective longitudinal studies are required to validate the causal relationship between these variables.

## 5. Limitations and Future Directions

This study has several limitations. The cross-sectional design precludes the establishment of causality. For instance, we are unable to distinguish whether lower gastrointestinal-specific anxiety serves as a cause or a consequence of high ARFID behaviors. Although mechanistic explanations grounded in the FAM were proposed and the stability of the High ARFID Symptom Risk Group (characterized by a high FRQoL-25 score, a low VSI, and full-time employment) was validated through sensitivity analyses (excluding patients with a disease duration of <1 year or recent relapses) the direction of causality still requires confirmation through longitudinal studies.

Three additional methodological limitations merit consideration. First, the single-center design with a predominance of Crohn’s disease may limit the generalizability of the findings. The clinical complexity inherent to such patients could lead to an overestimation of the proportion of individuals falling into the High ARFID Symptom Risk Group. However, the primary objective of this study was not to estimate prevalence rates but to explore the heterogeneous structural patterns of ARFID behaviors among patients with IBD in remission. Future multicenter, prospective studies are needed to validate the stability of these subgroups.

Second, the exclusive reliance on self-report scales introduces the potential for common method bias. Although instruments with established reliability and validity were employed and Harman’s single-factor test yielded negative results, this potential bias cannot be entirely ruled out. To further assess the robustness of our key findings, we examined the discriminant validity between distinct psychological constructs (e.g., the VSI vs the PHQ-4 anxiety subscale), confirming that while correlated, they remain conceptually distinct. More importantly, the core finding of this study, the paradoxical profile of “subjectively feeling well yet exhibiting high behavioral risk,” stems from a divergence between the results of different instruments (the NIAS, which assesses behaviors, vs the FRQoL-25/VSI, which assess subjective feelings). This divergence mitigates, to some extent, the issue of common method bias. Future studies incorporating behavioral observations or clinical interviews could provide more robust evidence.

Third, the absence of DSM-5-aligned structured clinical interviews for ARFID means that all findings are based on the NIAS, a validated screening tool for ARFID symptom risk. Therefore, our results are specific to ARFID symptom risk rather than confirmed clinical diagnoses. Future research should incorporate gold-standard diagnostic assessments to validate the clinical significance of the identified latent groups.

## 6. Conclusion

Patients with IBD in remission exhibit distinct classification characteristics regarding ARFID. Health care professionals should prioritize identifying patients with varying risk profiles during clinical assessments. Particular vigilance is required for high-behavioral-risk individuals who report a positive subjective sense of well-being characterized by low visceral sensitivity anxiety and a high food-related quality of life and who hold full-time employment; this is to prevent potential patterns of dietary avoidance and restriction from being overlooked because of these patients’ positive self-reports. In terms of management, a combined approach involving behavioral screening and structured dietary interviews should be adopted to implement targeted nutritional education and psychological interventions. The aim is to increase patients’ awareness of dietary behavioral risks and improve their dietary flexibility, thereby optimizing long-term nutritional outcomes. Considering the single-site nature of the current investigation’s participant recruitment, the external validity of its conclusions warrants careful consideration. Subsequent studies would benefit from broadening data collection to multiple sites and incorporating longitudinal methodologies. Such an approach would enhance the capacity to more robustly substantiate potential causal links between diverse risk factors and the manifestation of ARFID symptomatology.

## Acknowledgments

The authors thank all patients who participated in this study and the clinical staff of the Department of Gastroenterology, Nanjing First Hospital, for their support with participant recruitment and data collection.

## Author contributions

**Conceptualization:** Yuan Tian, Jimin Qiao.

**Data curation:** Yuan Tian, Jimin Qiao.

**Methodology:** Yuan Tian, Bo Gao, Jimin Qiao.

**Software:** Yuan Tian, Jimin Qiao.

**Writing – original draft:** Yuan Tian, Jimin Qiao.

**Writing – review & editing:** Yuan Tian, Bo Gao, Jimin Qiao, Huanhuan Fei, Yin Wu, Mengya Xu.

**Formal analysis:** Bo Gao.

**Supervision:** Bo Gao.

**Investigation:** Huanhuan Fei, Yin Wu, Mengya Xu.

**Project administration:** Huanhuan Fei, Yin Wu, Mengya Xu.
